# Prediction of the Medial Meniscal Size Using Radiograph Measurement of the Medial Proximal Surface of the Tibia in Dogs

**DOI:** 10.1111/vru.70053

**Published:** 2025-06-17

**Authors:** Simone Scherer, Kauê Danilo Helene Lemos dos Reis, Bernardo Schmitt, Alessandra Ventura da Silva, Marcelo Meller Alievi

**Affiliations:** ^1^ Department of Animal Medicine College of Veterinary Medicine Federal University of Rio Grande do Sul Porto Alegre Brazil

**Keywords:** medial meniscal dimension, meniscal transplantation, stifle, tibial plateau

## Abstract

Using radiographic measurements to predict meniscus size can enhance presurgical meniscus transplantation planning. Therefore, we aimed to predict the size of the medial meniscus through radiographic measurement of the medial proximal surface of the tibia. Twenty‐four pelvic limbs from medium and large breed dog cadavers had radiographs, and measurements were made from the tibial plateau length in mediolateral 90° and 135° stifle positions (TPL ML90 and TPL ML135) and medial tibial condyle width in the craniocaudal position (MTCW CrCd). The menisci were brushed with a positive contrast mixture, and radiographs were repeated at the same positions. The radiographic brushed meniscus was measured for each length position (CMML ML90, CMML ML135) and width position (CMMW CrCd). The anatomic size (the actual meniscus) was measured for the width (AMW) and the length (AML) and was compared with the radiograph after contrast was added to the meniscus. The ratio (%) of the positive contrast meniscus and conventional radiographic measurements in all positions were ML90 = 83.4 ± 6.9%, ML135 = 85.3 ± 8.11%, and CrCd = 97.9 ± 5.1%. Linear regression analysis equations based on the radiographs measurement values predicted (*p *≤ .01) the size of the meniscus (CMML ML90 = 9.7+0.433×TPL ML90; CMML ML135 = 11.7+0.371×TPL ML135; and CMMW CrCd = 2.9+0.775×MTCW CrCd). The intraclass correlation coefficient was used to quantify the agreement between AMW and AML with contrasted radiograph measurements [0.89 for CMMW CrCd (*p *< .01), 0.91 for CMML ML90 (*p *< .001), and 0.89 for CMML ML135 (*p *< .001)]. In conclusion, the width and length of the medial meniscus can be predicted based on the radiographic tibial plateau and medial tibial condyles measurements.

## 1 Introduction

The stifle is a frequently affected joint in dogs, and its most common pathology is cranial cruciate ligament instability (CrCLI). It was reported that 10 to 70% of dogs diagnosed with CrCLI had an associated meniscal lesion [1]. Larger dogs are more likely to be affected by CrCLI. In a study with 104 dogs (117 stifles), 97 stifles presented meniscus injuries. Of these dogs, 63.2% weighed more than 30 kg, and 36.7% weighed up to 30 kg [2]. A usual treatment approach to meniscus injuries in veterinary medicine is the surgical removal of all or part of a torn meniscus (i.e., meniscectomy) [3, 4].

The menisci function is to maintain the integrity of the femorotibial joint, protecting the articular cartilage. There are ways to evaluate if this function is normal, for example, by measuring the changes in femorotibial contact areas and contact pressure between joint surfaces. Mechanical pressure of the femorotibial contact has been evaluated in different types of partial meniscectomies of the caudal pole of the medial meniscus in dogs. The authors reported that in meniscectomies of 30% of the meniscus area, there were no significant changes in the biomechanics of the meniscus on its surface, but in meniscectomies of 75% of the meniscus area, there were significant changes in the biomechanical function of the meniscus and the mechanics of femorotibial contact [5]. For this reason, maintaining a greater residual volume of healthy meniscus was important for adequate contact pressure into the stifle joint [5].

Alternatives to meniscectomy have been studied in veterinary medicine. For instance, three kinds of meniscal sutures were biomechanically evaluated, and the stifle contact pressure was successfully restored, which is promising to be an alternative to partial meniscectomy in dogs [6]. Others have reported success in the functional healing and biomechanical integrity of the meniscus by testing a conduit device. This apparatus has a central axial channel to provide a potential site for the migration of cells and blood vessels for avascular meniscal defects in dogs [7].

In humans, meniscus transplantation is an alternative to meniscectomy, where the repair of meniscus injuries and partial meniscectomy are not possible [8]. Dogs have been used as experimental models in meniscus transplants and for testing different compounds to replace natural meniscus [9]. In dogs, information regarding the utility of meniscus transplantation is sparse [10]. Studies usually do not consider the size match of the meniscus since they are aimed at humans and not dogs [7]. However, the success of meniscus transplantation largely depends on the size compatibility of the meniscus between the donor and recipient [9]. A mismatch in graft selection of less than 10% of the size of the original meniscus may be acceptable and allow for the function of the meniscus to be restored [11]. Measurement methods are necessary to obtain the correct meniscus size to be transplanted and avoid its extrusion [10].

Sizing of the meniscus can be performed using radiographs, CT, and MRI in humans [10, 12]. In dogs, prediction of the meniscus size has been reported using the correlation of morphometric measurements [10] and magnetic resonance imaging of the meniscal volume in 1.5T MRI with a 3.0T system [13]. However, the experiment did not provide a valid measurement technique. There is a lack of scientific investigation in determining the size of the medial meniscus in dogs. Therefore, it is crucial to establish reliable measurement methods of the menisci in dogs to investigate, more accurately, meniscus transplant techniques, as well as their outcomes and methodology. In the present study, we aimed to develop and validate an equation to determine the measurements of the medial meniscus of medium to large dogs from measurements done in radiographs.

## 2 Materials and Methods

### 2.1 Selection and Description of Subjects

All experimental procedures were approved by the Federal University of Rio Grande do Sul (UFRGS, Brazil) Ethics Committee on the Use of Animals (protocol number 30069). Frozen dog cadavers were sourced from the Veterinary College Hospital Pathology and Town Shelter (Porto Alegre, RS, Brazil) and selected by an experienced orthopedic veterinary surgeon. A total of twenty‐four pelvic limbs from twelve cadavers of adult dogs were used. Medium to large‐sized cadavers had an estimated minimum weight of 20 kg. There was no sexual predilection or breed. Pelvic limbs with arthrosis and juvenile animals with evidence of open physes or conformational alteration were excluded from this study. The sample size was determined based on the variance (standard error) for the measurement of interest that was reported by other authors [6]. The dog cadavers were received frozen with no weight records. Nonetheless, femur radiographs were performed to guarantee the dog cadavers belonged to the same size group of medium to large dogs as described [14]. In brief, the length of the femur in the CrCd radiograph was measured from the intertrochanteric fossa to the distal margin of the femoral intercondylar fossa, parallel to the femoral axis. The proximal limit was on a transverse line from the most proximal point of the femoral head, and the distal limit was on a transverse line from the most distal end of the femoral condyles [14]. After the radiographs, the pelvic limbs were disarticulated, stored in plastic bags, numbered, and frozen at –20°C for further analysis.

### 2.2 Data Recording and Analysis

Frozen limbs were thawed for approximately 15 h. They were submerged in water at room temperature before their manipulation. Conventional radiographs of the tibia, including femorotibiopatellar and tibiotarsal joints of the 24 limbs at mediolateral positioning in 90° stifle flexion (ML90), mediolateral positioning in 135° stifle flexion (ML135), and craniocaudal positioning (CrCd) were performed. Special attention was given to the positioning of the tibia for the radiographs. The radiographic positioning was made by overlapping the tibial condyles for the correct measurement of the tibial plateau. The radiograph positions were chosen since they are commonly used for tibial plateau level osteotomy surgical planning in cases of CrCLI in dogs. A sphere of known size was kept close to the stifle joint to be used as a correction factor for the image magnification. The X‐ray beam was centered on the stifle joint. The entire tibia and tarsocrural joint were positioned directly over the radiographic cassette. The focal distance between the anode and the cassette was set at 100 cm, and the radiographic technique range was 65–70 KVp, 200 mA, and 0.03 s on a computed radiography (CR) system.

**FIGURE 1:**
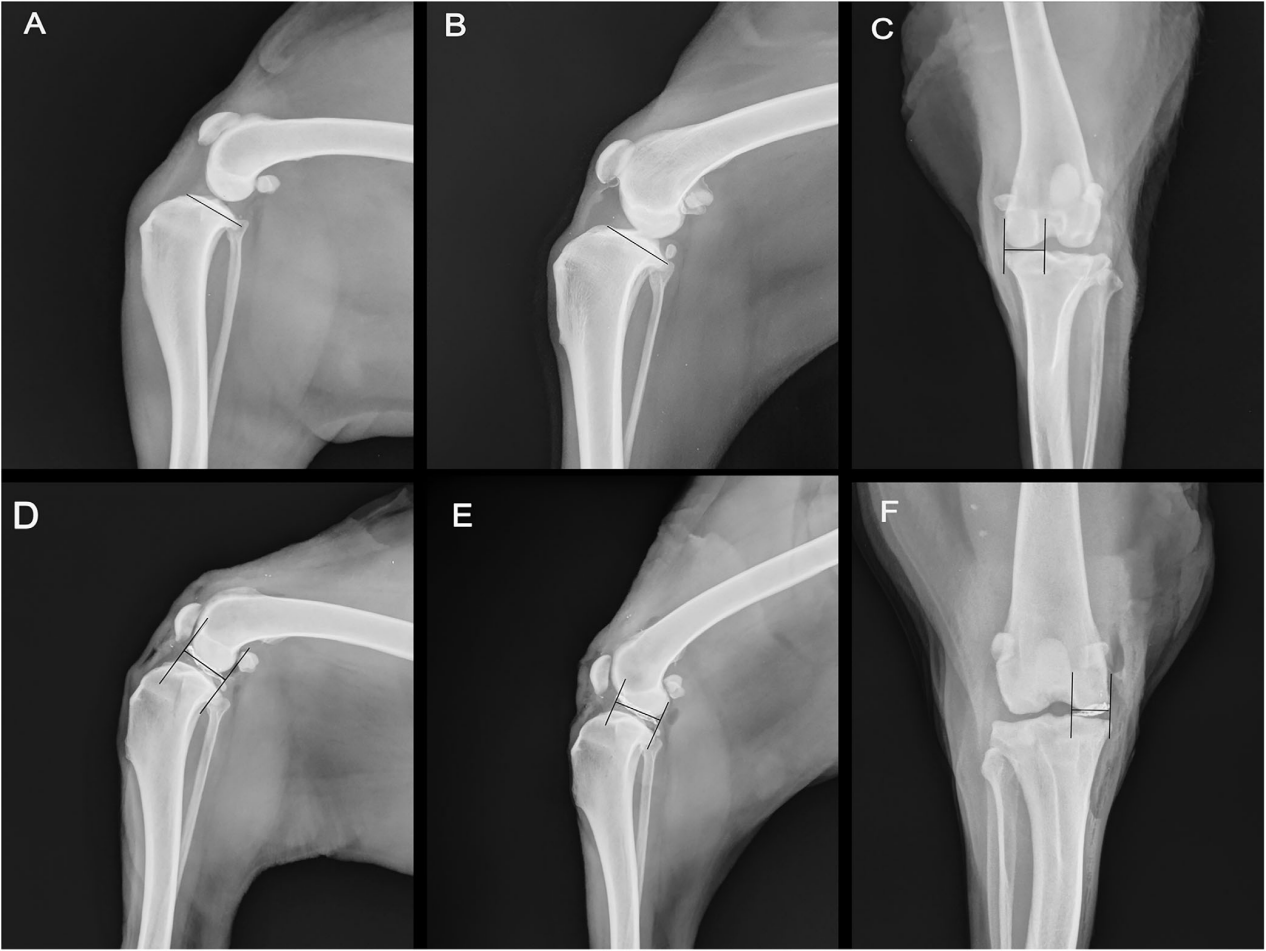
Measurement on simple radiographs TPL ML90 (A), TPL ML135 (B), and MTCW CrCd (C). Measurement of the contrasted Meniscus in CMML ML90 (D), CMML ML135 (E), and CMMW CrCd (F). Abbreviations: TPL ML90, tibial plateau length measurement in mediolateral 90° radiograph positioning; TPL ML135, tibial plateau length measurement in mediolateral 135° radiograph positioning; MTCW CrCd, medial tibial condyle width measurement in craniocaudal radiograph positioning; CMML ML90, contrasted medial meniscus length measurement in mediolateral 90° radiograph positioning; CMML ML135, contrasted medial meniscus length measurement in mediolateral 135° radiograph positioning; CMMW CrCd, contrasted medial meniscus width measurement in craniocaudal radiograph positioning.

Radiographs were acquired using the Siemens Multix B—Polymat S (Siemens Healthcare Diagnósticos Ltda., Joinville, Brazil) equipment, and the digital image radiographs were processed on AGFA CR30‐X (Agfa Brazil, São Paulo, Brazil) dedicated workstation. An experienced veterinary orthopedic surgeon used radiographic equipment (VierView, AGFA) to perform measurements in DICOM images. One observer performed all the measurements. Each measurement was replicated twice, and the mean value was used as an outcome. For the pelvic limbs, a CrCd projection was acquired to measure the distance between the medial limit of the tibia proximal medial to the medial intercondylar eminence. The measurement was named the medial tibial condyle width measurement (MTCW CrCd). The tibial plateau was measured in positions ML90 and ML135 and determined by the most cranial and most caudal limits of the tibial surface. The measurement was named tibial plateau length measurement (TPL ML90 and TPL ML135) (Figure 1A–C).

**FIGURE 2:**
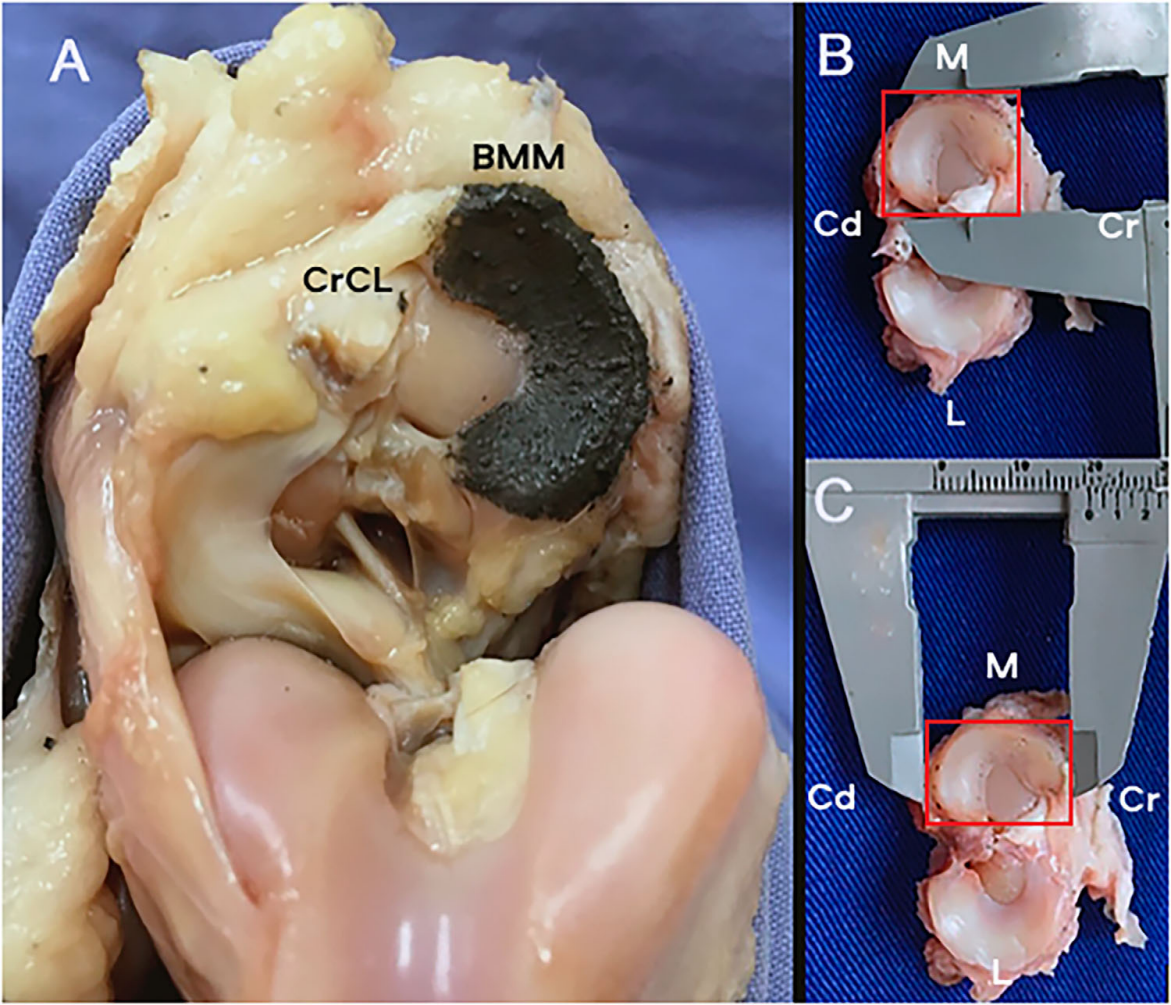
A, Medial meniscus bushed with a mixture of cyanoacrylate and tantalum in an open femorotibiopatellar joint. B, Measurement of the medial meniscus with a caliper as if it were inside an imaginary rectangle (red line), which represents anatomic meniscal width (B) and anatomic meniscal length (C). Abbreviations: CrCL, cranial cruciate ligament; BMM Brushed medial meniscus; Cr, cranial; Cd, caudal; M medial; L lateral.

After radiographs, a medial arthrotomy of the femorotibiopatellar joint was performed by an experienced orthopedic veterinary surgeon. The menisci were exposed completely by an incision of the medial collateral, patellar, cranial, and caudal cruciate ligaments, and the joint capsule in its medial caudal aspect, close to the medial meniscus. The tissue present around the edges of the menisci was gently dissected, and the medial meniscus was brushed with a mixture of cyanoacrylate and tantalum powder 325 (99.9%) in the proportion of two parts of cyanoacrylate to one part of powdered tantalum (Figure 2A). After the mixture dried, the femoral condyles were repositioned, and the patellar and medial collateral ligaments were sutured with Sultan pattern, nonabsorbable monofilament thread 2–0 (Technofio, Goiania, Brazil). The joint capsules were sutured with a simple continuous suture pattern and nonabsorbable monofilament thread 2–0 (Technofio). The subcutaneous tissue and skin were sutured with the same pattern and thread but with a thickness of 3–0.

**FIGURE 3:**
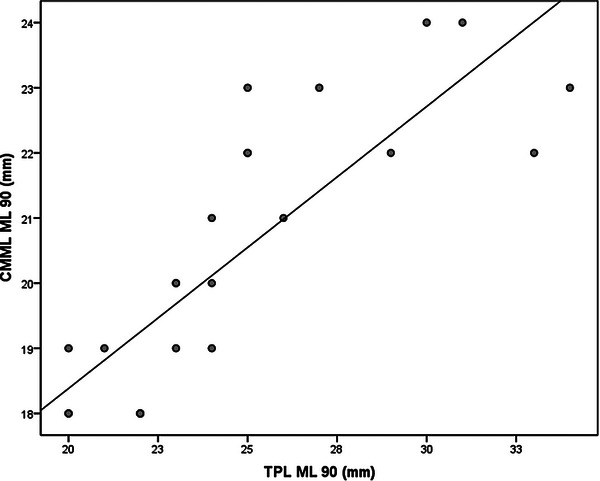
Scatterplot of simple linear regression from CMML ML 90 and TPL ML 90. CMML ML 90, contrasted medial meniscus length in mediolateral 90 positioning; TPL ML 90. Tibial Plateau Length in mediolateral 90 positioning.

**FIGURE 4:**
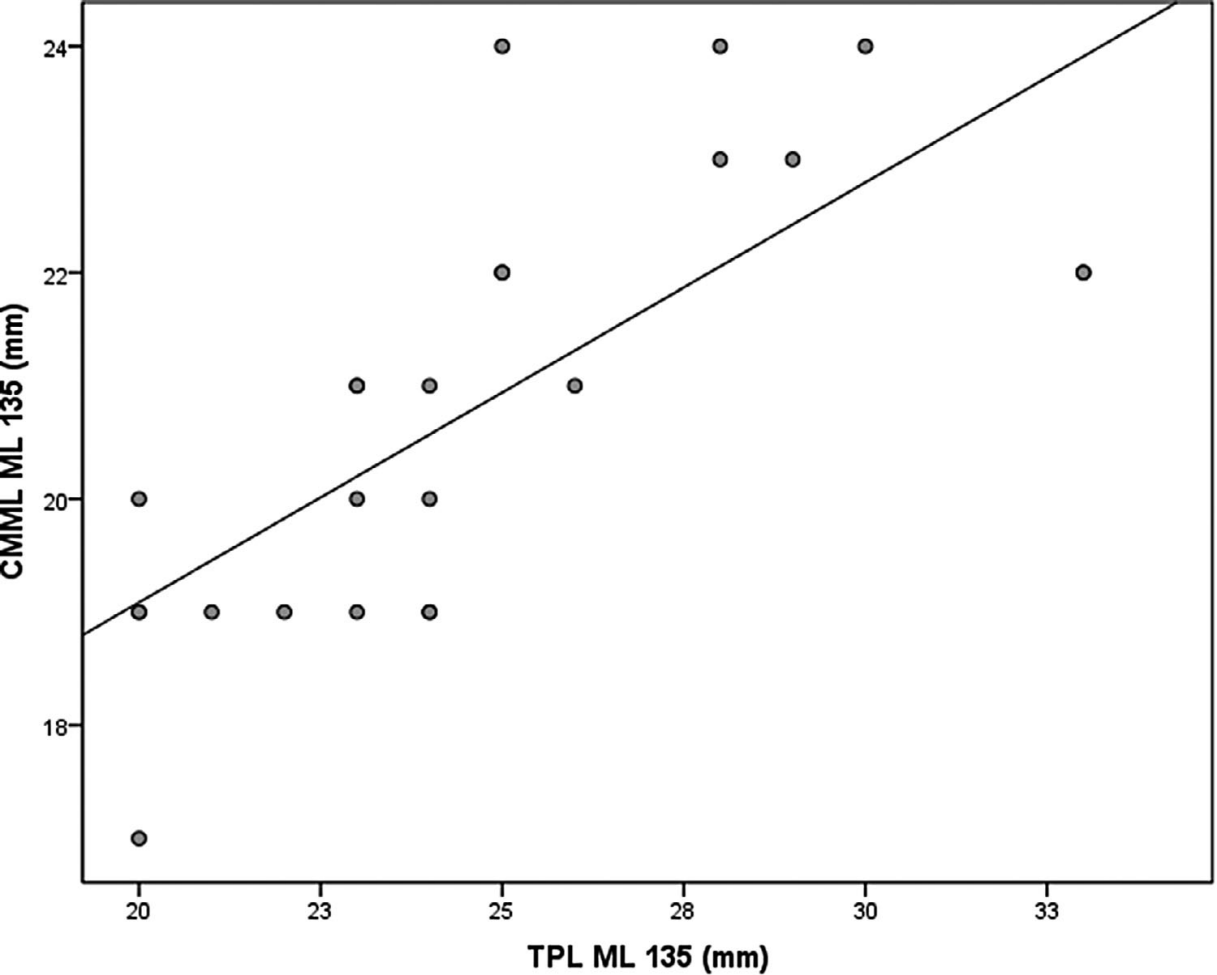
Scatterplot of simple linear regression from CMML ML 135 and TPL ML 135. Abbreviations: CMML ML 135, contrasted medial meniscus length in mediolateral 135 positioning; TPL ML 135. Tibial Plateau Length in mediolateral 135 positioning.

Radiographs were performed again in the CrCd, ML90, and ML135 positions as described previously. The metal opacity region on the radiograph, which represents the brushed medial meniscus, was measured. Two parallel lines were traced on the limits of the contrasted line. The measurement was performed from one line to the other. This measurement was performed in all radiographic projections. From these, two lateral contrasted medial meniscus length measurements were obtained: named CMML ML90 and CMML ML135, as well as the contrasted medial meniscus width measurement named CMMW CrCd (Figure 1D–F). The lateral contrasted measurements represented the length of the medial meniscus, and the craniocaudal contrasted measurements represented the width of the medial meniscus.

The joints were accessed again, and the medial menisci were measured with a manual caliper (Stafer Ferramentas, Porto Alegre, Brazil). The measurements were performed as if they were inside a rectangle whose medial side was the most medial part of the insertion of the meniscal ligament, and the lateral side was the point of the most lateral rim of the medial meniscus. The cranial limit was determined by the most cranial area, and the caudal limit was determined by the most caudal area. The measurement between the cranial and caudal edges of the rectangle was established as the anatomic meniscal length (AML) (Figure 2C). The measurement between the lateral and medial edges of the rectangle was used as the anatomic meniscal width of the medial meniscus (AMW) (Figure 2B). The radiographic measurement values were rounded to the nearest tenth.

### 2.3 Statistics

**FIGURE 5:**
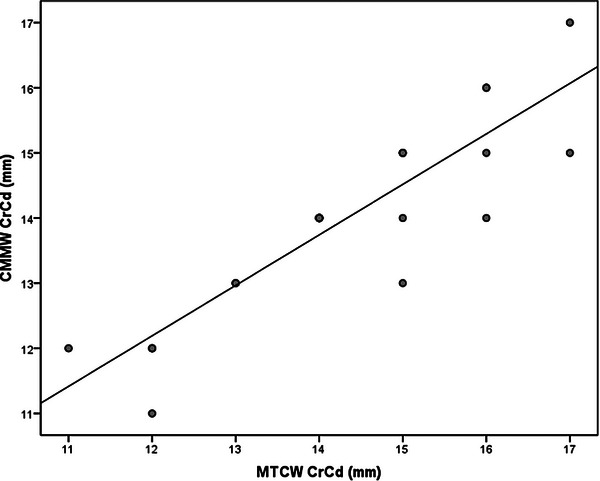
Scatterplot of simple linear regression from CMMW CrCd and MTCW CrCd. Abbreviations: CMMW CrCd, Contrasted medial meniscus width in craniocaudal position; MTCW CrCd, Medial tibial condyle width in craniocaudal positioning.

Statistical analysis was performed using the SPSS version 18.0. (Inc. Released 2009. PASW Statistics for Windows, Chicago: SPSS Inc., USA) software by a professional statistician. The estimation of values for the reference population was performed using a 95% confidence interval. Normality was determined by the Shapiro‐Wilk normality test. The mean values and standard deviation for each conventional and contrasted radiograph measurement were calculated in ML90, ML135, and CrCd positioning and anatomic meniscal measurements. A paired *t*‐test was performed to compare the right and left limbs and to compare conventional and contrasted radiographs at positionings ML90 and ML135. The ratio of the contrasted brushed menisci radiograph measurements to the conventional radiograph measurements for ML90, ML135, and CrCd positioning was expressed as a percentage (proportion) of the mean value of measurements. A simple linear regression analysis was used to predict the size of the meniscus based on the radiograph measurement values, in all radiograph positioning. The three linear regression analysis dependent variables were CMML ML90, CMML ML135, and CMMW CrCd, and the independent variables were TPL ML90, TPL ML135, and MTCW CrCd, respectively. The linear regressions and the intercept and slope were reported for each regression analysis (Table 2). Additionally, scatterplots for each linear regression were included (Figures 3, 4, 5).

Intraclass correlation coefficient (ICC) was used to quantify the agreement between the AML and CMML ML90, CMML ML135 measurements, and AMW and CMMW CrCd measurements. Statistical significance for all analyses was declared at *p* ≤ .05.

## 3 Results

Of the twelve dog cadavers used, eight were mixed‐breed dogs, one was a Great Dane, one was a Rottweiler, one was a Labrador retriever, and one was a Brazilian Fila. The length of the femur in the CrCd positioning was (mean ± SD) 166.7 ± 18.0 mm.

The use of a contrast mixture in a proportion of two parts of cyanoacrylate to one part of powdered tantalum proved to be efficient in showing the meniscus on radiographs. However, there was a break in the film applied on three stifles during the radiographic positioning maneuver of ML90, where the film needed to be removed and brushed again.

The mean values, standard deviation, and confidence interval of the anatomic menisci for conventional and contrasted menisci radiograph measurements are in Table 1.

**TABLE 1:** Mean values, standard deviation, and confidence interval of the anatomic radiograph measurements with and without contrast (values in mm).

Measurement	Mean ± SD (*n* = 24) (mm)	95% Confidence interval (mm)
TPL ML 90	24.8 ± 4.0	23.1–26.5
CMML ML 90	20.5 ± 2.1	19.6–21.3
TPL ML 135	24.7 ± 3.8	23.1–26.3
CMML ML135	20.8 ± 1.9	20.0–21.7
AML	20.3 ± 2.0	19.4–21.1
MTCW CrCd	14.3 ± 1.6	13.7–15.0
CMMW CrCd	14.0 ± 1.4	13.4–14.6
AMW	14.8 ± 1.4	14.2–15.4

Abbreviations: AMW anatomical meniscal width; CMML ML135, Contrasted medial meniscus length measurement in mediolateral 135° radiograph positioning; CMML ML90, contrasted medial meniscus length measurement in mediolateral 90° radiograph positioning; CMMW CrCd, contrasted medial meniscus width measurement in craniocaudal radiograph positioning, AML, anatomic meniscal length; MTCW CrCd, medial tibial condyle width measurement in craniocaudal radiograph positioning; SD, standard deviation. TPL ML135, tibial plateau length measurement in mediolateral 135° radiograph positioning; TPL ML90, tibial plateau length measurement in mediolateral 90° radiograph positioning.

There were no differences between the values obtained for the right side of the limbs in comparison with the left side (*p* > .05). Each stifle was used as a separate sample.

There was a difference between the values obtained in the contrasted menisci radiographs CMML ML90 and CMML ML135 (20.5 ± 2.1 and 20.8 ± 1.9, respectively; *p* = .009). However, there was no difference between the measurements of radiographs TPL ML90 and TPL ML135 (24.8 ± 4.0 and 24.7 ± 3.8, respectively; *p* = .679).

The lateral radiograph measurements between conventional and contrasted brushed menisci showed differences for both angles, TPL ML90 and CMML ML90 (4.333 mm) and TPL ML135 and CMML ML135 (3.875 mm) (*p* < .001 for both comparisons). The measurements of craniocaudal positioning between MTCW CrCd and CMMW CrCd had a small difference (0.333 mm) (*p* = .043).

The ICC between the contrasted menisci radiographs measurements and anatomic menisci measurements were 0.89 for CMMW CrCd and AMW (*p* < .01), 0.91 for CMML ML90 and AML (*p* < .001), and 0.89 for CMML ML135 (*p* < .001).

The ratio of the contrasted menisci radiograph measurements to the conventional radiograph measurements were (mean ± SD) 83.4 ± 6.9% for the ML90 positioning, 85.3 ± 8.11% in the ML135 positioning, and 97.9 ± 5.1% in the CrCd positioning. The linear regression equations are in Table 2. The scatter plots are in Figures 3, 4, 5.

**TABLE 2:** Linear regression equations to obtain the anatomic meniscal width and length applied in ML and CrCd radiographs measurements (mm).

Meniscal dimensions Length ML90‐135 Width CrCd (mm)	Formula using radiograph measurements of TP on ML 90, ML135 radiographs positions, and MTC in CrCd radiograph position (mm)
CMML ML 90=	9.7 + 0.433 × TPL ML 90 (*R* ^2^ = 0.69; *p* ≤ .01)
CMML ML 135=	11.7 + 0.371 × TPL ML 135 (*R* ^2^ = 0.53; *p* ≤ .01)
CMMW CrCd=	2.9 + 0.775 × MTCW CrCd (*R* ^2^ = 0.77; *p* ≤ .01)

Abbreviations: ML, mediolateral; CrCd, craniocaudal; TP, tibial plateau; MTC, medial tibial condyle; CMML ML135, contrasted medial meniscus length measurement in mediolateral 135° radiograph positioning; CMML ML90, contrasted medial meniscus length measurement in mediolateral 90° radiograph positioning; CMMW CrCd, contrasted medial meniscus width measurement in craniocaudal radiograph positioning; MTCW CrCd, medial tibial condyle width measurement in craniocaudal radiograph positioning; TPL ML135, tibial plateau length measurement in mediolateral 135° radiograph positioning; TPL ML90, tibial plateau length measurement in medio lateral 90° radiograph positioning.

## 4 Discussion

Our hypothesis was supported in that we reported equations that satisfactorily predicted the anatomic meniscal measurements from conventional radiographs of medium‐ and large‐breed dogs.

The measurements performed on the femur of the specimens in the present study agree with the classification from other authors [14]. These authors aiming to get precise values of femoral neck angle in support of developing a hip prosthesis, used dogs of medium and large breeds and allocated them into two groups according to the femoral length: Group I (from 145 to 195 mm) and Group II (from 196 to 240 mm). The dogs which belonged to group I had an average body weight of 27 kg (17 to 45 kg). The dogs of medium to large body weight in the present study had breed representativity of mixed breed dogs.

Researchers [15, 16] have mainly reported on Labrador retrievers CrCLI rather than mixed breed dogs. One could conclude that Labradors are more prone to meniscal injury. However, we believe it is important to consider similar studies in specific breeds and sizes of dogs with more representativity, which could cause certain biases in the results.

In the present work, the wide joint access made it possible to brush the meniscus with the mixture in dog cadavers, as proposed by a study in humans [17]. However, unlike the 1:1 ratio of the mixture used by these authors, we used the proportion 2:1 ratio of cyanoacrylate to powdered tantalum (99.9%) to delay drying and facilitate the brushing of the menisci. Breakage of the mixture film was observed in three cases, and it was not reported by the aforementioned authors. Most likely, the conformation of the dog's stifle was associated with greater pressure in the caudal region of the medial meniscus when performing the ML90 positioning, which may have favored the breakage of the film. We elected to perform the mediolateral measurement at two different angles, ML90 and ML135, which is different from that performed in a similar study in humans [17, 18]. In that study, the authors had the human's leg in an extended position in the lateral radiographs. Our approach aimed to verify if there would be a difference in the values of the menisci in different stifle joint angulations that are frequently used for radiograph exploration and surgical planning of the stifle in dogs [19].

We did not observe differences between the measurements of lateral radiographs of TP (TPL ML90 and TPL ML135). However, there were differences between the lateral positioning of contrasted medial menisci radiograph measurements (CMML ML90 and CMML ML135). That can be explained by the compression of the femoral condyle on the medial meniscus during joint flexion. In veterinary practice, it is important to apply the correct equation for each position performed since there is the possibility of surgery to be planned according to the meniscus size. No differences between the measurements of the right and left limbs in all positions for conventional and contrasted radiographs were found. However, other authors [20, 21] reported a difference in meniscal dimensions between the right and left limbs in humans, demonstrating anatomic variability between the menisci of each leg in the same person. This deserves attention in the case of using the meniscal measurements of the contralateral limb to determine the size of the meniscus in humans. Future research is needed to better understand the fitment of the measurements presented in this study when dogs are affected by pathologies in one of the limbs. For instance, severe degenerative joint disease (DJD) or other changes that make it difficult to obtain clearly caudal TP limit in conventional radiographs. Therefore, severe DJD may be a limitation for the use of these measurements from radiographs.

One study [22] evaluated the observer variability of tibial slope measurements and correlations with the degree of DJD at the caudal joint border in dogs. There was interobserver variation in the measurements, and no significant differences were associated with DJD. However, the most variability was related to the cranial and caudal points of selection. The DJD obscured the caudal point, especially. Another study [23] in small dogs aimed to describe a method to perform TP measurement using CT and compare it with the conventional radiograph method in terms of landmark identification. The authors described an easier identification of the anatomic landmarks of TP on CT. Compared with the radiographic method, measurements using CT were subjectively easier for the identification of landmarks and also showed higher intra‐ and interobserver agreement. These results suggest that the use of CT to measure TP in cases of severe DJD in dogs is beneficial. The same author suggested the use of a rotating 3D image to correct the position for measurement instead of repeating the radiographs. In this study, we did not evaluate joints with DJD, but we acknowledge the potential differences and need to establish a pattern for menisci measurement; it is necessary to carry out further studies to determine the best option to measure the TP in dogs with severe DJD.

We did not account for differences in radiograph measurements due to sex. Other researchers [11] reported on the relationship between morphologic characteristics and meniscus size in dogs and concluded that there was a difference of 0.6 mm in width and 0.4 mm in length for females who had smaller meniscal measurements when compared with male dogs. In humans, it was reported [24] that the correlation of the meniscal size, gender, height, and weight for men and women had different intercondylar notch widths, with women generally having smaller total tibial plateau widths than men. Therefore, there is a need for further research on meniscal size and sex in dogs.

In the present work, the anatomic meniscal measurements were quite difficult to obtain because the cranial and caudal poles of the menisci are not aligned and not collinear in a pure sagittal plane. Drawing a rectangle aligned with an axial line intersecting the most medial fibrocartilage of both the cranial and caudal meniscal poles was critical for measuring the width and length of the meniscus [11, 17]. Others [20] have reported in humans on measurements of the meniscal size by measuring at the peripheral‐most point of the inner, free margin of each meniscus. The width of the medial and lateral menisci from the attachment of the posterior horn to the outermost edge of the peripheral rim was determined by the circumference of the medial and lateral meniscus. In the present study, we measured the anatomic width and length of the medial meniscus using a rectangular shape even though the rectangle was not aligned with the caudal and cranial insertion poles of the meniscus ligaments (Figure 2B,C). In this way, the meniscus was measured the same as the view of the brushed meniscus position CrCd and ML in the radiograph.

A study [17] on the human knee suggested the meniscus size by establishing the ratio (percentage) of the radiographs of tibial plateau measurements and the radiographs of brushed meniscus measurements. They determined the extent to which the brushed meniscus was depicted in relation to the tibial plateau in both mediolateral and anteroposterior radiograph positions. They inferred that the medial meniscus length represented 82% of the ML position of the TP measurement and the width, 98% in the anteroposterior position measurement of the tibial surface. Similarly, in the present study, we were able to estimate the length and width of the meniscus through the percentual of the radiograph measurements of the brushed meniscus over the measurements of the radiographs of the TPL ML90, TPL ML135, and MTCW CrCd.

Through linear regression, equations were obtained in such a way that radiograph measurements were able to predict the meniscal width and length satisfactorily in the present study (Table 2; Figures 3, 4, 5). Other researchers [20] used linear regression analysis on anatomic menisci and anatomic TP in human cadavers. The authors reported good predictability for the lateral width, fair for lateral length and medial width, and poor for medial length menisci.

A small difference (less than 1 mm) was observed between medium sizes of contrasted brushed menisci radiographs and actual menisci in length and width. These exceeded values of imaging measurements occurred due to residual magnification, despite of magnification correction carried out. Research on the human knee pointed to a positive image absolute difference in radiography in width 1.95 ± 1.43 mm and length 2.75 ± 2.05 mm [21]. We did not evaluate meniscus shrinkage in this study, and we believe this deserves further attention when studying the application (surgery technique) of our results.

The ICC in the present study suggests that the values are in good and excellent agreement. The contrasted brushed menisci radiographs represent a good agreement of anatomic meniscal dimensions in CMML ML90 and CMML ML135 and an excellent agreement in CMMW CrCd measurements, different from other studies that reported poor agreement in some of the compared parameters [18, 20].

Predictions vary due to differences between the measurements performed radiographically instead of anatomically [20]. For instance, authors [18] have reported that the measurements for the length of the lateral tibial plateau by angling the radiographic beam 10° caudally at a neutral rotation, allowed differentiation of the lateral plateau cortical margins from the medial plateau in humans. The transition points were identified and used for length measurement. However, in dogs, positioning of the tibia during radiography may influence the appearance of anatomic landmarks and the tibial plateau, which may vary according to the rotation and the inclination when the radiographs are acquired [19].

In the surgical practice of meniscal allograft transplantation in humans, it is common to determine the desired meniscal size by measuring the width or the length alone, not simultaneously. One study [18] on human menisci reported that the relation between the anatomic width and length had a poor linear correlation (*R* ^2^ = 0.425, *p* = .241). In the present study, a much stronger linear correlation for CMMW CrCd (*R* ^2^ = 0.77; *p* ≤ 0.01) and a stronger linear correlation for CMML ML90 (*R* ^2^ = 0.69; *p* ≤ .01), and a good linear correlation in CMML ML 135 (*R* ^2^ = 0.53; *p* ≤ .01) was reported. Therefore, it is important to measure both the width and the length and prepare the allograft for proper sizing [18]. Additionally, others have reported a poor correlation (*R* ^2^ < 0.5) among the meniscal height, width, and length [25]. In conclusion, findings from our study contribute to the body of literature on the dimensions of the menisci in medium to large dogs. More importantly, the percentual and predictive linear equations allowed for the prediction of the meniscus size in dogs using radiographs. This information is of utmost importance in veterinary medicine as it advances the field toward meniscus transplant in dogs.

## Conflicts of Interest

The authors declare no conflicts of interest.
